# The Role of HIV-Related Stigma in Utilization of Skilled Childbirth Services in Rural Kenya: A Prospective Mixed-Methods Study

**DOI:** 10.1371/journal.pmed.1001295

**Published:** 2012-08-21

**Authors:** Janet M. Turan, Abigail H. Hatcher, José Medema-Wijnveen, Maricianah Onono, Suellen Miller, Elizabeth A. Bukusi, Bulent Turan, Craig R. Cohen

**Affiliations:** 1 Department of Health Care Organization and Policy, School of Public Health, University of Alabama at Birmingham, Birmingham, Alabama, United States of America; 2 Department of Obstetrics, Gynecology, and Reproductive Sciences, University of California, San Francisco, San Francisco, California, United States of America; 3 University of Groningen, Groningen, The Netherlands; 4 Centre for Microbiology Research, Kenya Medical Research Institute, Nairobi, Kenya; 5 Department of Psychology, University of Alabama at Birmingham, Birmingham, Alabama, United States of America; Massachusetts General Hospital, Harvard Medical School, United States of America

## Abstract

**Background:**

Childbirth with a skilled attendant is crucial for preventing maternal mortality and is an important opportunity for prevention of mother-to-child transmission of HIV. The Maternity in Migori and AIDS Stigma Study (MAMAS Study) is a prospective mixed-methods investigation conducted in a high HIV prevalence area in rural Kenya, in which we examined the role of women's perceptions of HIV-related stigma during pregnancy in their subsequent utilization of maternity services.

**Methods and Findings:**

From 2007–2009, 1,777 pregnant women with unknown HIV status completed an interviewer-administered questionnaire assessing their perceptions of HIV-related stigma before being offered HIV testing during their first antenatal care visit. After the visit, a sub-sample of women was selected for follow-up (all women who tested HIV-positive or were not tested for HIV, and a random sample of HIV-negative women, *n* = 598); 411 (69%) were located and completed another questionnaire postpartum. Additional qualitative in-depth interviews with community health workers, childbearing women, and family members (*n* = 48) aided our interpretation of the quantitative findings and highlighted ways in which HIV-related stigma may influence birth decisions. Qualitative data revealed that health facility birth is commonly viewed as most appropriate for women with pregnancy complications, such as HIV. Thus, women delivering at health facilities face the risk of being labeled as HIV-positive in the community. Our quantitative data revealed that women with higher perceptions of HIV-related stigma (specifically those who held negative attitudes about persons living with HIV) at baseline were subsequently less likely to deliver in a health facility with a skilled attendant, even after adjusting for other known predictors of health facility delivery (adjusted odds ratio = 0.44, 95% CI 0.22–0.88).

**Conclusions:**

Our findings point to the urgent need for interventions to reduce HIV-related stigma, not only for improving quality of life among persons living with HIV, but also for better health outcomes among all childbearing women and their families.

*Please see later in the article for the Editors' Summary.*

## Abstract 2

Janet Turan and colleagues examined the role of the perception of women in rural Kenya of HIV-related stigma during pregnancy on their subsequent utilization of maternity services.

## Editors' Summary

**Background:**

Every year, nearly 350,000 women die from pregnancy- or childbirth-related complications. Almost all these “maternal” deaths occur in developing countries. In sub-Saharan Africa, for example, the maternal mortality ratio (the number of maternal deaths per 100,000 live births) is 500 whereas in industrialized countries it is only 12. Most maternal deaths are caused by hemorrhage (severe bleeding after childbirth), post-delivery infections, obstructed (difficult) labor, and blood pressure disorders during pregnancy. All these conditions can be prevented if women have access to adequate reproductive health services and if trained health care workers are present during delivery. Notably, in sub-Saharan Africa, infection with HIV (the virus that causes AIDS) is an increasingly important contributor to maternal mortality. HIV infection causes maternal mortality directly by increasing the occurrence of pregnancy complications and indirectly by increasing the susceptibility of pregnant women to malaria, tuberculosis, and other “opportunistic” infections—HIV-positive individuals are highly susceptible to other infections because HIV destroys the immune system.

**Why Was This Study Done?:**

Although skilled delivery attendants reduce maternal mortality, there are many barriers to their use in developing countries including cost and the need to travel long distances to health facilities. Fears and experiences of HIV-related stigma and discrimination (prejudice, negative attitudes, abuse, and maltreatment directed at people living with HIV) may also be a barrier to the use of skilled childbirth service. Maternity services are prime locations for HIV testing and for the provision of interventions for the prevention of mother-to-child transmission (PMTCT) of HIV, so pregnant women know that they will have to “deal with” the issue of HIV when visiting these services. In this prospective mixed-methods study, the researchers examine the role of pregnant women's perceptions of HIV-related stigma in their subsequent use of maternity services in Nyanza Province, Kenya, a region where 16% women aged 15–49 are HIV-positive and where only 44.2% of mothers give birth in a health facility. A mixed-methods study combines qualitative data—how people feel about an issue—with quantitative data—numerical data about outcomes.

**What Did the Researchers Do and Find?:**

In the Maternity in Migori and AIDS Stigma (MAMAS) study, pregnant women with unknown HIV status living in rural regions of Nyanza Province answered questions about their perceptions of HIV-related stigma before being offered HIV testing during their first antenatal clinic visit. After delivery, the researchers asked the women who tested HIV positive or were not tested for HIV and a sample of HIV-negative women where they had delivered their baby. They also gathered qualitative information about barriers to maternity and HIV service use by interviewing childbearing women, family members, and community health workers. The qualitative data indicate that labor in a health facility is commonly viewed as being most appropriate for women with pregnancy complications such as HIV infection. Thus, women delivering at health facilities risk being labeled as HIV positive, a label that the community associates with promiscuity. The quantitative data indicate that women with more negative attitudes about HIV-positive people (higher perceptions of HIV-related stigma) at baseline were about half as likely to deliver in a health facility with a skilled attendant as women with more positive attitudes about people living with HIV.

**What Do These Findings Mean?:**

These findings suggest that HIV-related stigma is associated with the low rate of delivery by skilled attendants in rural areas of Nyanza Province and possibly in other rural regions of sub-Saharan Africa. Community mobilization efforts aimed at increasing the use of PMTCT services may be partly responsible for the strong perception that delivery in a health facility is most appropriate for women with HIV and other pregnancy complications and may have inadvertently strengthened the perception that women who give birth in such facilities are likely to be HIV positive. The researchers suggest, therefore, that health messages should stress that delivery in a health facility is recommended for all women, not just HIV-positive women or those with pregnancy complications, and that interventions should be introduced to reduce HIV-related stigma. This combined strategy has the potential to increase the use of maternity services by all women and the use of HIV and PMTCT services, thereby reducing some of the most pressing health problems facing women and their children in sub-Saharan Africa.

**Additional Information:**

Please access these Web sites via the online version of this summary at http://dx.doi.org/10.1371/journal.pmed.1001295.

- The United Nations Children's Fund (UNICEF) provides information on maternal mortality, including the WHO/UNICEF/UNFPA/World Bank 2008 country estimates of maternal mortality; a UNICEF special report tells the stories of seven mothers living with HIV in Lesotho
- The World Health Organization provides information on maternal health, including information about Millennium Development Goal 5, which aims to reduce maternal mortality (in several languages); the Millennium Development Goals, which were agreed by world leaders in 2000, are designed to eradicate extreme poverty worldwide by 2015
- Immpact is a global research initiative for the evaluation of safe motherhood intervention strategies
- Maternal Death: The Avoidable Crisis is a briefing paper published by the independent humanitarian medical aid organization Médecins Sans Frontières (MSF) in March 2012
- Information is available from Avert, an international AIDS charity on all aspects of HIV/AIDS, including information on women, HIV and AIDS, on HIV and pregnancy, on HIV and AIDS stigma and discrimination, and on HIV in Kenya (in English and Spanish); Avert also has personal stories from women living with HIV
- The Stigma Action Network (SAN) is a collaborative endeavor that aims to comprehensively coordinate efforts to develop and expand program, research, and advocacy strategies for reducing HIV stigma worldwide, including mobilizing stakeholders, delivering program and policy solutions, and maximizing investments in HIV programs and services globally
- The People Living with Stigma Index aims to address stigma relating to HIV and advocate on key barriers and issues perpetuating stigma; it has recently published Piecing it together for women and girls, the gender dimensions of HIV-related stigma
- The Health Policy Project http://www.healthpolicyproject.com has prepared a review of the academic and programmatic literature on stigma and discrimination as barriers to achievement of global goals for maternal health and the elimination of new child HIV infections (see under Resources)
- More information on the MAMAS study is available from the UCSF Center for AIDS Prevention Studies

## Introduction

Each year, approximately 342,900 maternal deaths occur globally, the vast majority in developing countries [1]. Among these, an estimated 42,000–60,000 maternal deaths were attributed to HIV infection in 2009 [2]. Recent data have shown that HIV is an increasing contributor to direct and indirect causes of maternal deaths in sub-Saharan Africa [3]. Evidence-based strategies are urgently needed to reduce the global burden of pregnancy-related deaths, particularly in resource-constrained settings [4].

Increasing skilled delivery attendance is one evidence-based strategy for improving maternal and infant health for all women [5],[6]. Skilled delivery attendance can also enhance the effectiveness of interventions for the prevention of mother-to-child transmission (PMTCT) of HIV, as women who give birth with the assistance of a health professional are more likely to receive information about and adhere to antiretroviral drugs [7],[8]. However, many barriers to women's utilization of skilled delivery attendance have been identified globally, including cost and distance to health facilities [9], fears of harsh treatment [10], low educational levels, low incomes, lack of women's empowerment [11], and long-standing cultural traditions of delivering at home [12].

In addition to these relatively well-known impediments to pregnant women's utilization of health services in low-resource settings, there is growing evidence that fears and experiences of HIV-related stigma pose an added barrier. Qualitative studies in sub-Saharan Africa have suggested that pregnant women avoid health clinics if they fear HIV testing or unwanted HIV status disclosure [13]–[15]. Indeed, stigma and discrimination have been revealed as among the most important barriers to pregnant women's acceptance of HIV testing during antenatal care [16]–[18], their initial participation in programs for PMTCT [19],[20], and their retention and adherence in these programs [21]–[23].

What is less clear is the extent to which HIV-related stigma shapes women's decisions around use of skilled childbirth services. It is recognized that different dimensions of HIV-related stigma may have separate adverse effects on quality of life, health care access, and health outcomes for persons living with HIV (PLWH) [24], and some research suggests that stigma from close persons may have a bigger impact [25],[26]. Anticipated stigma refers to a person's fears of stigma and discrimination if other people would learn about his/her HIV status [27], while measures of perceived community stigma capture societal norms and values, as well as perceptions about the ways that PLWH are treated in the broader community [28]. In addition to having a profound impact on the mental health and quality of life of PLWH [29]–[31], HIV-related stigma can also indirectly worsen health when fears of stigma, shame, or denial cause avoidance of essential health services [32].

Qualitative studies suggest that for pregnant women in high HIV-prevalence settings, HIV-related stigma—especially from a woman's male partner—may be a key factor causing women to avoid antenatal clinics (ANC) and childbirth in health facilities [33],[34]. Maternity services have become prime locations for HIV testing and provision of PMTCT interventions in Kenya and many other countries. Thus, all pregnant women become aware that they will have to “deal with” the issue of HIV when visiting maternity services, and in rural Kenya many women perceive that they will be forced to test for HIV at health facilities [35].

As Kenya has made only limited progress towards reaching the maternal and child health Millennium Development Goals [36], understanding women's use of skilled delivery and HIV-related health services is an urgent priority. In Nyanza Province, Kenya, an estimated 16% of women aged 15–49 are HIV-positive, more than double the national average [37]. While 93.6% of pregnant women access some antenatal care, only 44.2% deliver their babies in a health facility [37]. Traditional birth attendants (TBAs) are active in the province, assisting in an estimated 26% of births [37]. However, most do not have any formal training and the Kenyan health authorities discourage their practice. In this context of high HIV prevalence and low skilled birth attendance, HIV-related stigma may have negative effects on all women, not just those who are aware that they are HIV-infected. In this paper, we examine the role of women's perceptions of HIV-related stigma during pregnancy in their subsequent utilization of skilled childbirth services.

## Methods

### Ethics Statement

This study received ethical approval from the Kenya Medical Research Institute (KEMRI) Ethical Review Committee, the University of California, San Francisco Committee on Human Research, and the University of Alabama at Birmingham Institutional Review Board.

### Study Design

We conducted a prospective, mixed-methods study to examine the impact of HIV-related stigma on skilled delivery utilization among pregnant women. In the Maternity in Migori and AIDS Stigma Study (the MAMAS Study), we followed pregnant women from the time of their first ANC visit up to 4–8 wk postpartum. Using “best practice” techniques for mixed-methods health research [38], the MAMAS Study collected quantitative data to provide measurable evidence of the impact of HIV-related stigma on skilled delivery attendance, alongside qualitative data to understand why and how this dynamic manifests itself in the study communities. The MAMAS Study sites were government health facilities receiving support from the Family AIDS Care and Education Services (FACES) Program for HIV prevention, care, and treatment.

At baseline, pregnant women unaware of their current HIV status (never tested or tested negative more than 3 mo ago) were recruited, were taken through a signed informed consent process, and then completed a structured questionnaire—comprising mostly close-ended questions—that had been programmed on a personal digital assistant (PDA) [39]. This baseline questionnaire was administered by a trained interviewer immediately prior to the woman's first ANC visit. The consent process was carried out orally using simple wording in the language preferred by the participant (Dholuo, Swahili, or English) and included special procedures to ensure understanding and actual informed consent for potential participants who could not read or write. Additional inclusion criteria for the baseline “early pregnancy questionnaire” included being 18 y of age or older and gestational age of 28 wk or less. Subsequently, all pregnant women were routinely offered voluntary HIV counseling and rapid testing as part of the ANC visit as per national guidelines and, if they consented, information on their acceptance of HIV testing and their HIV serostatus were obtained from their medical records after the visit. Recruitment and baseline questionnaires for the MAMAS Study (*n* = 1,777) took place between November 2007 and April 2009. Women also provided consent for subsequent follow-up and contact information at the time of the baseline questionnaire.

For follow-up, we selected all women who tested HIV-positive, all those who were not tested for HIV at the first ANC visit, and a random sample of women who tested HIV-negative. To balance these groups by site and over time, approximately every 2 wk during the baseline data collection period at each clinic we selected for follow-up all the women who tested HIV-positive, all the women who were not tested for HIV (refused or were not offered an HIV test), and a random sample of women who tested HIV-negative equal to the number of HIV-positive women (using a computer-based random number generator). This resulted in a sample of 598 women selected for follow-up (226 HIV-positive, 227 HIV-negative, and 145 HIV status unknown). Of those 598 women, 411 women (69%) could be located and participated in a postpartum questionnaire either at the health facility (12%) or at their home (88%), administered by a trained interviewer using a structured questionnaire in local languages (English, Dholuo, or Swahili) programmed on a personal digital assistant (PDA). Women who completed the questionnaires received a small monetary reimbursement for travel expenses related to study participation for each questionnaire (equivalent of around US$3); an amount judged by the KEMRI ethical review committee to be fair but not coercive. Given the poverty of the rural area where the research was conducted, even the small amount of money provided was an extra incentive for people to participate in the study. The fact that women of all HIV status categories were included in the research (HIV-negative, HIV-positive, and HIV status unknown) reduced the possibility that participation in the research itself would be stigmatizing.

In order to gain more in-depth understanding of the barriers to maternity and HIV service use at the community level, we also conducted qualitative research in four communities in the study districts. In each community, we purposively sampled from several key informant groups: four pregnant or postpartum women who did not use facility-based maternity services, three TBAs, three male partners or other family members of pregnant/postpartum women, and two community health workers, for a total of 48 in-depth interviews. These in-depth interviews were conducted in local languages by experienced Kenyan qualitative interviewers (age and gender appropriate) using open-ended qualitative interview guides developed by the research team. Interviews generally lasted 1–2 h and were audio-recorded after obtaining the written informed consent of the participant, using similar informed consent procedures to those described above. This portion of the study was introduced in the community through meetings and discussions with community leaders and local health workers. These interviews were not exclusively focused on HIV and participants did not have to be HIV-positive or disclose their HIV status to participate. Privacy was maintained as much as possible by allowing participants to select a private location in the community where others could not observe or hear the interview. Similar to the questionnaires, participants received a small monetary reimbursement (equivalent of around US$3) for travel expenses.

### Measures

#### Outcome

The primary outcome of interest was the woman's self-reported location where she gave birth for her most recent pregnancy. Based on women's responses, we created a binary variable for giving birth in the health facility or outside the health facility. Since skilled attendants do not assist births outside of the health facility in Kenya, we considered giving birth in a health facility to be equivalent to giving birth with a skilled attendant (although recognizing that the quality of birth attendance provided in health facilities may vary [40]).

#### HIV status

The woman's HIV test result was obtained from her medical record after the initial ANC visit. During follow-up questionnaires, women who were HIV-negative or HIV status unknown at the first visit were asked to report any subsequent HIV testing and results. In follow-up questionnaires, 13 additional women reported an HIV-positive test result.

#### HIV-related stigma and discrimination measures

Baseline perceptions of HIV-related stigma and discrimination were measured using multi-item scales demonstrated to be valid and reliable in sub-Saharan African settings [16]. Anticipated HIV-related stigma (fears of stigma for self if one was to test HIV-positive and others found out) was measured using a nine-item scale originally developed and tested in a study in Botswana [41]. Reliability of this total score was high in our sample (Cronbach's α = 0.86). Variables were created to examine anticipated stigma by source; we created dichotomous measures of anticipated stigma from the male partner (answered yes to either or both of the male partner items), from family members (answered yes to either or both of the family items), and from others (answered yes to one or more of the items regarding others).

To measure perceived community stigma, we used the 22-item stigma scale developed by National Institute of Mental Health (NIMH) Project Accept [28], with higher scores representing increased stigma. The scale had a high degree of internal consistency in our sample (Cronbach's α = 0.85) and assesses three dimensions: the participant's own negative attitudes and beliefs about PLWH (negative attitudes, eight items; α = 0.78), perceptions of acts of discrimination faced by PLWH within the community (discrimination, seven items; α = 0.75), and attitudes and beliefs related to fair treatment of PLWH (equity, four items; α = 0.70). Mean sub-scale scores were computed for women who had no more than one missing item in that sub-scale, and the mean total score was computed only for those who had valid scores on all three sub-scales. Possible mean scores on the scales ranged from 0 to 3; with negative items reverse-coded so that higher scores indicated higher perceptions of HIV-related stigma. In the sample for the current analyses, total scale scores ranged from 0 to 2.39 (mean = 0.86, standard deviation [SD] = 0.36), with the negative attitudes sub-scale scores ranging from 0 to 2.86 (mean = 0.80, SD = 0.45), the perceived discrimination sub-scale scores ranging from 0 to 2.17 (mean = 0.91, SD = 0.38), and the equity scores ranging from 0 to 3.00 (mean = 0.87, SD = 0.57). The mean sub-scale score for negative attitudes towards PLWH in our sample is similar to mean scores found in South Africa (0.79–0.93), but lower than the mean found in Tanzania (1.66), while the mean sub-scale score for perceived discrimination in our sample is lower than those found in both South Africa (1.76–2.02) and Tanzania (2.04) [28].

#### Other potential predictors

Other predictors of utilization of skilled childbirth services were selected using a socio-ecological approach to understanding the inter-related factors that influence women's use of facility-based labor and delivery services [42],[43]. We examined individual socio-demographic characteristics, such as age, education, marital status, parity, ethnicity, and indicators of household wealth. We measured health care factors shown to influence skilled birth attendance in other studies [10],[33],[44], including: use of antenatal care, distance to the health facility, expectations regarding costs, and perceptions of the quality of care. Lastly, we measured relationship factors, including family decision-making processes about the place of delivery, involvement of the woman's male partner [45], and experience of intimate partner violence (physical, sexual, or psychological) during pregnancy or postpartum.

#### Key concepts explored in the qualitative interviews

Topic areas that we identified for exploration in qualitative interviews included gender roles and decision-making around reproductive health, roles of TBAs, barriers to using facility-based maternity services, motivators for using maternity services, effects of fear of HIV-related stigma on health service use, and issues surrounding disclosure of HIV-positive status.

### Analytical Methods

#### Quantitative

We initially compared the socio-demographic characteristics of women who could be located and participated in the postpartum questionnaire with those who were lost to follow-up using statistical tests for categorical (chi-square or Fisher's exact tests) and continuous variables (*t*-tests or Kruskal-Wallis tests). Next we examined the associations of women's HIV status (HIV-positive, HIV-negative, or unknown) with measures of perceived community stigma and anticipated stigma at baseline using ANOVA and chi-square tests. The next step was to examine the bivariate and multivariate associations of potential predictor variables with the main outcome, delivery at a health facility, using logistic regression methods. In order to account for the fact that women were recruited in eight different clinics, we used random effects models [46] with clinic as the cluster variable using Stata 11 (StataCorp).

#### Qualitative

Audio files from the in-depth interviews were transcribed in the original language (Dholuo, Swahili, or English) and then translated to English by experienced trilingual translators based in Kenya. The English transcripts were coded by two researchers (AHH, JMW) in QSR NVivo, using a thematic approach to data analysis [47]. Initial broad coding was guided by major themes from the interview guides, but new codes and themes were developed on the basis of the data. Excerpts and analytical memos were reviewed by two authors (JMT, AHH) to identify common themes and variant views. During a second round of analysis, the initial broad codes were further detailed into sub-themes using a grounded theory approach to developing fine codes [48]. The definitions of these emerging fine codes were then discussed and refined among the research team. After finalizing the coding scheme, printed reports of each fine code were used to write a detailed analytical report (JMW, AHH). Illustrative quotations that most clearly represent each theme were chosen to be included in the manuscript.

#### Synthesis of quantitative and qualitative methods and data

During data collection, we used initial findings from quantitative structured questionnaires to inform subsequent in-depth qualitative interview topics. This is considered a “sequential” data collection approach, where one method informs data collection of the other [49]. During data analysis, however, we integrated the quantitative and qualitative data, allowing each set of findings to deepen, enrich, and complement the other [50]. To accomplish this, we first prepared separate preliminary reports on quantitative and qualitative findings. For each thematic area, we examined both the quantitative and the qualitative data to elucidate the findings that emerged from each method and to identify areas where the findings were in congruence, diverged, or added insight to one another. This approach ultimately provided a richer set of conclusions, and helped triangulate findings that may have been difficult to interpret on their own [51]. Though mixed methods research continues to evolve, one might call this a “pure mixed interpretative” approach, since we placed equal weight on both qualitative and quantitative findings [52], and the main point of interface between methods was at the data interpretation stage [53].

## Results

Women who were located and participated in a postpartum questionnaire (*n* = 411, 69%) were similar in socio-demographics to women who were lost to follow-up, except for household radio ownership (those followed up more likely to own, *p* = 0.002). 30% of women lost to follow-up were in a polygynous relationship (husband had other wives), compared to 24% of women who participated postpartum (*p* = 0.105). The two groups did not differ significantly in terms of HIV status after the first ANC visit (Table 1).

**Table 1:** Comparison of baseline socio-demographic characteristics of MAMAS Study participants followed up after the birth versus lost to follow-up (*n* = 598).

Variables^a^	Followed up Postpartum (*n* = 411)	Lost to Follow-up Postpartum (*n* = 187)	Statistical Test Result
**Mean age**	23.8 (23, 18–40)	23.6 (22, 18–48)	t = −0.257, *p* = 0.797
**Education, ** ***n*** ** (%)**			
Primary school or less	355 (86.4)	151 (80.7)	χ^2^ = 3.12, *p* = 0.077
More than primary	56 (13.6)	36 (19.3)	
**Religion**			
Roman Catholic	70 (17.0)	41 (21.9)	χ^2^ = 2.34, *p* = 0.311
Seventh Day Adventist	131 (31.9)	60 (32.1)	
Other	210 (51.1)	80 (46.0)	
**Ethnicity**			
Luo	382 (92.9)	178 (95.2)	χ^2^ = 1.09, *p* = 0.297
Other	29 (7.1)	9 (4.8)	
**Household goods**			
Electricity	12 (2.9)	7 (3.7)	χ^2^ = 0.28, *p* = 0.594
Television	40 (9.7)	19 (10.2)	χ^2^ = 0.03, *p* = 0.871
Mobile phone	195 (47.4)	81 (43.3)	χ^2^ = 0.88, *p* = 0.348
Radio	323 (78.6)	125 (66.8)	χ^2^ = 9.43, *p* = 0.002
**Woman's occupation**			
Housework	92 (22.4)	41 (21.9)	χ^2^ = 0.38, *p* = 0.945
Selling things/fish monger	104 (25.4)	45 (24.1)	
Farming/agricultural/manual	174 (42.4)	80 (42.8)	
Other	40 (9.8)	21 (11.2)	
**Marital status**			
Not currently married	51 (12.4)	31 (16.7)	χ^2^ = 1.96, *p* = 0.162
Currently married	360 (87.6)	155 (83.3)	
**Living with male partner**			
Yes	355 (86.4)	155 (83.3)	χ^2^ = 0.95, *p* = 0.329
No	56 (13.6)	31 (16.7)	
**Polygynous household**			
Yes	98 (23.8)	56 (30.1)	χ^2^ = 2.62, *p* = 0.105
No	313 (76.2)	130 (69,9)	
**Male partner's occupation (** ***n*** ** = 509)** b			
Selling things	39 (11.0)	14 (9.0)	χ^2^ = 1.04, *p* = 0.903
Farming/agricultural work	115 (32.5)	56 (36.1)	
Fishing	61 (17.2)	24 (15.5)	
Manual labor	58 (16.4)	26 (16.8)	
Other type of job	81 (22.9)	35 (22.6)	
**Mean months of pregnancy** c	5.10 (5, 1–8)	5.07 (5, 2–7)	t = −0.320, *p* = 0.749
**Mean number of pregnancies**	3.18 (3, 1–10)	3.03 (2, 1–10)	t = −0.878, *p* = 0.380
**Mean number of live births**	2.13 (2, 0–9)	1.97 (1, 0–9)	t = −0.948, *p* = 0.343
**Mean number of living children**	1.84 (2, 0–9)	1.62 (1, 0–7)	t = −1.62, *p* = 0.106
**HIV status at first ANC visit**			
HIV-positive	154 (37.5)	72 (38.5)	χ^2^ = 4.07, *p* = 0.254
HIV-negative	165 (40.1)	62 (33.2)	
Refused HIV test	52 (12.7)	33 (17.6)	
No HIV testing available	40 (9.7)	20 (10.7)	

a For continuous variables, the mean followed by the median and range in parentheses are presented. For categorical variables, *n*'s are given followed by percentages in parentheses.

b Data on this variable were available for women who reported currently living with a male partner.

c Woman's self report.

### HIV-Related Stigma

Pregnant women in the MAMAS Study reported high levels of HIV-related stigma at baseline, with more than two-thirds anticipating HIV-related stigma from some source if they were to test HIV-positive. Women who ended up testing HIV-negative reported the highest levels of negative beliefs about the rights of PLWH to fair treatment (equity sub-score) at baseline (*p* = 0.012) (Table 2).

**Table 2:** Baseline perceptions of HIV-related stigma by HIV status at the time of the postpartum questionnnaire (*n* = 411).

Stigma Measures	HIV-Positive (*n* = 165)	HIV-Negative (*n* = 195)	HIV Status Unknown (*n* = 51)	Statistical Test Result
**Perceived community HIV-related stigma at baseline**				
Total score	0.8461	0.8951	0.7683	*F* = 2.61, *p* = 0.075
Negative attitudes sub-scale score	0.7622	0.8488	0.7276	*F* = 2.39, *p* = 0.093
Perceived discrimination sub-scale score	0.9327	0.9058	0.8596	*F* = 0.73, *p* = 0.484
Equity sub-scale score	0.8369	0.9524	0.7050	*F* = 4.43, *p* = 0.012
**Anticipated HIV-related stigma at baseline**				
Any	112 (70.4)	119 (65.0)	32 (68.1)	χ^2^ = 1.14, *p* = 0.564
From male partner	47 (33.1)	75 (43.9)	23 (53.5)	χ^2^ = 7.02, *p* = 0.030
From family	62 (39.5)	68 (37.0)	17 (35.4)	χ^2^ = 0.36, *p* = 0.834
From others	98 (66.2)	116 (67.8)	28 (63.6)	χ^2^ = 0.30, *p* = 0.860

More women were tested for HIV during the interim between the first ANC visit and the postpartum questionnaire, resulting in more HIV+ women, more HIV-negative women, and fewer HIV status unknown women.

The qualitative data revealed a strong perception in the study communities that HIV is associated with promiscuity and immorality. A majority of respondents described how PLWH in this setting are viewed as “not good,” loose, or involved in prostitution.

*They don't want anyone to find out that they are sick. Instead they want to be known as good people. If people find out that she has that disease then they will say that she was a prostitute, the kind of person who will have sex with someone whenever she goes to the market or goes to run any errand.* (Pregnant Woman 9, Ndiwa)

Some participants noted that the community stigma associated with an HIV diagnosis may be particularly salient for pregnant women, who may be blamed not only for being HIV-positive, but also for placing the health of their child at risk.

*Someone can say that if so and so had known that she had the disease, why is she giving birth? Someone who has that disease of AIDS, she can give birth to a child who has it. I feel that she will have made miserable the life of that child.* (Female Relative 1, Ndiwa)

More than half of women who still had not been tested for HIV by the time of their postpartum questionnaire had anticipated male partner stigma at baseline (53.5%), compared to 43.9% of those who were tested and found to be HIV-negative, and 33.1% of those who were tested and found to be HIV-positive (33.1%) (χ^2^ = 7.02, *p* = 0.030). A health worker explained that male partners often get angry if women accept an HIV test at the clinic without prior discussion.

*There are men who don't like it when their women come to the clinic, and they do quarrel their wives if they heard that the women were screened for HIV. That is the main reason as to why some women still don't disclose to their husband when they come to the clinic and get an HIV test. Some men can even send their women away just because of that.* (Community Health Worker 1, Ongo)

### Factors Associated with Delivery in a Health Facility

Overall, 35% of women who participated in the postpartum questionnaire (*n* = 411) reported that they gave birth in a health facility. Of the women who reported giving birth at home (*n* = 261), 78% reported assistance from a TBA. Table 3 presents the bivariate associations of potential predictor variables with this outcome, accounting for clustering by clinic.

**Table 3:** Associations of predictor variables with place of delivery (unadjusted, accounting for clustering by site) (*n* = 407).

Variable	Delivered at Home (*n* = 266)	Delivered at a Health Facility (*n* = 141)	OR for Delivery at a Health Facility^a^	95% CI	*p*-Value
**Socio-demographic characteristics at baseline**					
Mean age	23.78	23.79	1.00	0.96–1.04	0.994
Higher education (more than primary), *n* (%)	22 (8.3)	35 (24.8)	3.50	1.89–6.49	<0.001
Currently married, *n* (%)	233 (87.6)	123 (87.2)	0.90	0.47–1.72	0.744
Living in a polygynous household, *n* (%)	71 (26.7)	26 (18,4)	0.62	0.37–1.05	0.077
First pregnancy, *n* (%)	38 (14.3)	39 (27.7)	2.18	1.29–3.70	0.004
Working in farming or manual labor, *n* (%)^b^	131 (49.2)	41 (29.3)	0.46	0.29–0.74	0.001
Luo ethnic group, *n* (%)	252 (94,7)	126 (89,4)	0.45	0.20–1.02	0.057
Household owns a mobile phone, *n* (%)	114 (42.9)	80 (56.7)	1.56	1.01–2.41	0.043
**HIV-related measures**					
HIV-positive status at T1, T2, or T3, *n* (%)	106 (39.8)	55 (39.0)	0.99	0.64–1.54	0.972
Mean anticipated stigma score (*n* = 385)^c^	0.2882	0.2784	0.74	0.35–1.59	0.442
Mean total perceived community stigma score (*n* = 390)^d^	0.8792	0.8180	0.47	0.24–0.95	0.035
Mean negative attitudes sub-scale (*n* = 395)	0.8250	0.7434	0.52	0.30–0.89	0.017
Mean perceived discrimination sub-scale (*n* = 393)	0.9089	0.9149	0.79	0.43–1.46	0.453
Any knowledge of mother-to-child transmission of HIV	228 (87.7)	123 (90.4)	1.19	0.58–2.43	0.641
**Other individual-level predictors**					
IPV during pregnancy or postpartum^e^					
Reported any type	79 (30.0)	36 (25.5)	0.56	0.32–0.98	0.034
Unknown	98 (37.6)	43 (30.5)	0.51	0.30–0.86	0.012
Four or more ANC visits, *n* (%)	96 (36.1)	86 (61.0)	2.48	1.58–3.89	<0.001
Mean distance to the HF in minutes	86.88	79.74	1.00	1.00–1.00	0.837
Expects to paying for supplies and medicine	175 (66.8)	111 (81.6)	1.87	1.06–3.29	0.029
Discussion of delivery with male partner	176 (66.7)	108 (77,1)	1.83	1.10–3.04	0.020
Pregnant woman decided on delivery alone	151 (57,0)	58 (41.1)	0.51	0.32–0.79	0.003

407 women had non-missing data for both baseline and postpartum follow-up variables.

a Estimates account for clustering by site (health facility), using random effects logistic regression.

b As compared to those working in housework, selling things (including fish), or other occupations (combined).

c Mean anticipated stigma score was computed for women who had non-missing data for at least six of the nine items.

d Mean perceived community stigma sub-scale scores were computed for women who had no more than one missing item in that sub-scale, and the mean total score was computed for those who do not have more than one item missing on any sub-scale.

e As compared to women who responded “no” to all intimate partner violence (IPV) questions (physical, psychological, or sexual abuse) regarding IPV during pregnancy and postpartum. Women who refused to answer or had missing data were placed in the unknown category.

OR, odds ratio.

#### Socio-demographic factors

Women were more likely to deliver in a health facility if they had higher education and greater household wealth (as indicated by ownership of a mobile phone) [54]. Women for whom this was the first pregnancy were more than twice as likely to deliver in a facility, whereas women in agricultural occupations were less likely than those primarily engaged in housework, selling things, or other occupations.

In qualitative interviews, most participants explained that wealth and occupation often influenced delivery decisions since hospitals demand complete payment at the time of the delivery. This was viewed as especially difficult for women whose agricultural income pays in seasonal installments.

*The other thing that can prevent me from going to the hospital to give birth is because they will demand cash money from you. But, the traditional birth attendants will sympathize with you and let you pay later when you have money. I like that because, at times, delivery can come when you are broke and penniless.* (Pregnant Woman 2, Ogwedhi)

Some participants also added that women of low socio-economic status fear bad treatment at health facilities. For example, pregnant woman explained that health workers often treated impoverished patients harshly and that facility-based delivery was more appropriate for richer, educated women.

#### Relationship factors

Several relationship factors were significantly associated with delivery at the health facility in quantitative analysis: having discussed the place of delivery with the male partner/husband; having decided on the place together with the husband or others (i.e., not deciding alone); and not having experienced intimate partner violence (IPV) during pregnancy or postpartum. The influence of the male partner was emphasized by many of the qualitative interview participants and emerged as an important factor for use of maternity services in qualitative analysis.

*My sister-in-law has five children. And out of these five children, for none of them did the mother visit the ANC clinics when she was expecting. And her husband even tries to stop the wife when he hears that she wants to visit the clinic. He thinks that it will make his wife or the remaining children die, or something bad might happen to his children.* (Family Member 1, Brother-in-law, Ongo)

#### Health care factors

Women who completed four or more ANC visits during pregnancy or expected to pay for supplies and medicines related to the delivery were more likely to deliver at a health facility. These findings align with the qualitative interviews, within which a common perception was that women were required to have an antenatal clinic card to deliver at the health facility. Though one pregnant woman wanted to give birth at the local health facility, she assumed that she would be denied admittance since she failed to attend prenatal visits.

*I recently saw a woman who went to deliver her baby at the hospital, I was impressed with the advantage of delivering at the hospital, I admired her…. But, the hospital cannot admit me when I go there now. Maybe if I develop a complication and then they will charge me highly. But, if it is a normal delivery then they cannot help you because you have not been going to clinic and you don't have a clinic card. They will not help you the way that they can help someone who has been going to the clinic [throughout pregnancy]; they will just abandon you there.* (Pregnant Woman 2, Ndiwa)

Similar to this pregnant woman, participants in every respondent group described facility-based childbirth as most appropriate for women with complications. In many interviews, the health facility was seen as a last-resort for women whose births were expected to be abnormal or especially risky. Many participants in the qualitative interviews associated the need for health facility delivery with both pregnancy complications and HIV:

*They should be encouraged to seek treatment if they have HIV, and again be encouraged to deliver at the hospital. Because at times delivery may come with complications, which can only be handled better at the hospital.* (Pregnant Woman 1 Ndiwa)

#### HIV-related stigma, discrimination, and violence

Some participants described cases in which male partners had negative and even violent reactions to HIV testing and services conducted within maternity services.

*There are those who normally chase away their wives saying that they should just go, because he already thinks that the child is also having the disease. He will threaten to beat you up so your heart will be troubled because you have the disease, you are pregnant, and the man has chased you to go back to your home, all those will be painful. There is one story I heard about …that a man beat up his pregnant wife recently when she went to the clinic and was found with the virus. (Pregnant Woman 9, Ndiwa)*

Narratives like this are likely to influence delivery decisions, especially since the health facility is perceived to focus on HIV testing. Several participants believed that HIV testing was a mandatory part of visits to the health facility.

*I have heard some say that when you come to the hospital they test [for HIV]. But when they visit the TBA, there are no such questions. So that is why sometimes women dodge the hospital and visit the TBA.* (Male Relative 1, Minyenya)

In quantitative analyses, women with higher overall perceptions of community stigma against PLWH at baseline (total score) and those who held more negative attitudes and beliefs about PLWH (sub-scale) had significantly lower odds of delivering in a health facility. On the other hand, delivery in a health facility did not differ significantly by the different measures of anticipated HIV-related stigma for self at baseline (before HIV testing), nor by HIV status on its own (34.2% HIV-positive, 35.7% HIV-negative, and 32.0% HIV status unknown).

### Multivariate Analysis of Delivery in a Health Facility

In order to create a parsimonious model given our limited sample size, we used substantive knowledge to reduce the number of variables to be included in our final multivariate model, while retaining key variables indicated by the literature and theory [55]. Age and number of pregnancies were highly correlated. Given that all of the women in the sample were in a relatively narrow reproductive age range (18–40 y), whether this was the woman's first pregnancy or not is likely to be more relevant for decisions regarding place of childbirth. Therefore we included first pregnancy (Y/N) but not age in the final model. We retained education since it is a key variable strongly associated with place of delivery in the literature [11]. We included living in a polygynous household as this has been shown to be associated with a variety of reproductive behaviors in sub-Saharan Africa [56]. We did not include marital status and ethnicity since almost all women in our sample were married and of Luo ethnicity. We chose women's own occupational status as the indicator of wealth, as the “ownership of household goods” variables referred to the whole household and might not indicate the woman's own access to goods. We retained both HIV status at follow-up and anticipated stigma at baseline in the final model because we wanted to elucidate the effects of women's current perceptions of HIV-related stigma, while controlling for these. We also included both sub-scale scores measuring the woman's current perceptions of HIV-related stigma—both her own negative attitudes towards PLWH and her perceptions of discrimination against PLWH in the community—as these were primary variables of interest for this study. We did not include knowledge of mother-to-child transmission (MTCT) of HIV since almost all women in the sample had basic knowledge of MTCT (around 90%). We did not include the intimate partner violence variable since a substantial number of women were in the “unknown” category for this variable. We included number of ANC visits since adequate utilization of ANC is a strong predictor of health facility delivery in the literature [57]. We also chose to retain a measure of distance from home to the health facility, since this can be a major barrier in this setting [58]. We elected not to include the “expects to pay for supplies and medicines” variable since it was highly correlated with educational level and might simply reflect women's correct knowledge of the requirements for facility birth. We selected the woman's discussion of plans for the birth with her husband, as this was a strong predictor of health facility birth in another study conducted in this specific part of Kenya [59]. Thus our final multivariate model included education, polygyny, first pregnancy, woman's occupation, number of ANC visits during pregnancy, discussion of the plans for the birth with the husband, distance to the health facility, baseline anticipated stigma score, the woman's own negative attitudes towards PLWH subscale score, the woman's perceptions of discrimination against PLWH in the community subscale score, and her HIV status at the time of the postpartum questionnaire.

In this multivariate model, education and antenatal care retained their strong significant associations, with women with more than primary education having 3.01 times higher odds of having utilized skilled delivery services than women with less education (*p* = 0.003), and women who had four or more ANC care visits during pregnancy having 2.42 times higher odds of having used this service than women who had fewer ANC visits (*p* = 0.001). Women who had discussed the place of delivery with their husband/male partner had 2.02 higher odds of having given birth in a health facility than those who said they had not discussed this issue with their husband (*p* = 0.023). The negative attitudes towards PLWH sub-scale stigma score also retained significance in the adjusted model (*p* = 0.020), with those women having more negative attitudes about PLWH being less likely to deliver in a health facility (Table 4).

**Table 4:** Multivariate logistic regression of HIV-related stigma measures and other individual-level predictor variables on delivery in a health facility (*n* = 381).

Variable	Number of Women in the Category (*n*) in This Model	Adjusted OR for Delivery in a Health Facility^a^	95% CI	*p*-Value
**Educational Level**				
Primary or less^c^	328			
More than primary	53	3.01	1.45–6.22	0.003
**Living in a polygynous household**				
No^c^	291			
Yes	90	0.79	0.43–1.44	0.442
**First pregnancy**				
No^c^	315			
Yes	66	1.21	0.62–2.38	0.574
**Woman's occupation**				
Other^c^	212			
Farming/agriculture	169	0.49	0.29–0.84	0.010
**Distance from home to health facility (minutes)**	381	1.00	1.00–1.01	0.541
**Baseline anticipated HIV-related stigma score**	381	1.11	0.46–2.69	0.808
**Number of ANC visits during pregnancy**				
Less than 4^c^	214			
Four or more	167	2.42	1.45–4.03	0.001
**Discussed place of delivery with husband**				
No^c^	107			
Yes	274	2.02	1.10–3.69	0.023
**Own negative attitudes towards PLWH score**	381	0.44	0.22–0.88	0.020
**Perceived discrimination against PLWH in the community score**	381	1.52	0.68–3.39	0.310
**HIV status at postpartum questionnaire**				
HIV-negative^c^	181			
HIV-positive	154	1.22	0.72–2.08	0.455
Unknown	46	1.02	0.46–2.29	0.958

*n* is less than 407 due to small numbers of missing values on individual variables.

a Estimates also account for clustering by site (health facility) as a random effect.

c Reference category.

OR, odds ratio.

### Delivery in a Health Facility Stratified by Postpartum HIV Status

In order to understand how perceptions of stigma might operate differently on use of facility-based delivery services for HIV-infected versus HIV-uninfected women, we examined the interaction of the negative attitudes toward PLWH score with HIV status. The interaction term was statistically significant when added to the multivariate model (*p* = 0.025). In stratified bivariate analyses only accounting for clustering, the odds ratio for delivery in a health facility associated with having negative attitudes toward PLWH was 0.40 for both HIV-negative women (95% CI = 0.19–0.88) and women of unknown HIV status (95% CI = 0.07–2.42), while the odds ratio was 0.77 for HIV-positive women (95% CI = 0.33–1.78).

## Discussion

Our findings suggest that HIV-related stigma plays an important role in the low rates of skilled delivery in rural Nyanza, Kenya. Our qualitative data revealed a strong perception that childbirth in a health facility was most appropriate for women with health complications like HIV. Communication campaigns and community mobilization efforts aiming to increase utilization of PMTCT services have tended to emphasize certain maternal and child health practices, including health-facility delivery, specifically for HIV-positive women [60]. This may have inadvertently strengthened the image of health facilities as being predominantly for women with health problems. Likewise, it may have strengthened the perception that women who give birth in the health facility are likely to be HIV-positive. Maternity services have now become closely associated with HIV and PMTCT, and the trend is likely to be strengthened as a result of the current push for integration of maternity and HIV services globally [61]. Our quantitative data revealed that women with higher perceptions of HIV-related stigma (specifically those who held negative attitudes about PLWH) were less likely to deliver in a health facility, even after adjusting for other known predictors of health facility delivery.

Examining the interactions between HIV status and negative attitudes revealed that the effect of “perceptions of HIV-related stigma” was stronger for HIV-negative women and women of unknown HIV status than for HIV-positive women. These results suggest that if a woman is HIV-negative and holds negative attitudes about PLWH, she might not want to deliver in a health facility in order to avoid the assumption in the community that she is HIV-positive. Women who have received an HIV-negative test result at their first ANC visit may now consider themselves “safe.” However, given the evidence of continuing high HIV-infection risk for women throughout pregnancy [62],[63], more efforts should be focused on HIV-related stigma and risk reduction counseling for women who test HIV-negative in ANC clinics.

For women who did test HIV-positive at the ANC clinic, other considerations—such as her perception of the risk of mother-to-child transmission of HIV or whether or not she has disclosed her HIV status to her husband and family—may play a bigger role. The importance of avoiding unwanted disclosure of HIV status in HIV-positive women's decisions about where to give birth has been suggested in other studies in Kenya [21],[33].

We also found support for several factors that have been shown to be related to women's use of facility-based childbirth services in cross-sectional studies in other settings [10],[11]. In analyses adjusted for other predictors we found that women with higher levels of education and women who had four or more antenatal care visits during pregnancy were more likely to deliver their babies in a health facility. We also found that involvement of the male partner in decision-making about the place of delivery was positively associated with health facility delivery. This is similar to findings of the Skilled Care Initiative Study conducted in nearby districts in rural Nyanza, in which husband involvement in decision-making was one of the factors most strongly associated with skilled care-seeking for childbirth [59].

### Strengths and Limitations

An important strength of this study is the examination of these issues in low-resource rural communities highly affected by HIV, rather than in controlled urban research clinics, which makes the results more likely to be generalizable to rural settings where the majority of women live in sub-Saharan Africa. In addition, the prospective design allowed us to quantitatively assess women's fears and perceptions of HIV-related stigma at baseline during pregnancy, and then follow them prospectively to learn about their subsequent maternity service utilization. We were able to use a variety of validated measures of HIV-related stigma and provide quantitative evidence for effects of HIV-related stigma on health care utilization that have been suggested by a wealth of qualitative studies [13],[64],[65]. In fact, this is one of the first studies to quantitatively show an effect on HIV-related stigma on pregnant women's use of health services. Finally, our mixed methods research design allowed us to both examine quantitative associations among variables and to explore the motivations and reasons behind those associations in an in-depth manner.

Limitations of the study included the loss to follow-up in our sample between the early pregnancy and postpartum questionnaires. Although the rate of loss to follow-up was around 30%, we could not detect any significant biases (except for household radio ownership) in those who were located and participated postpartum versus those who were not. However, it is possible that the women lost-to-follow up were different in ways that we did not measure. Another limitation of the quantitative study is that it included only women who came for at least one ANC visit during the first 28 wk of pregnancy and who were at least 18 y of age. Women who did not come for any ANC visits or who came for their first ANC visit late in pregnancy may be affected by HIV-related stigma and discrimination in different ways. Women under age 18 were not included in the study because of the difficulties of obtaining informed consent for minors in this setting, but we also recognize that the relationships between HIV-related stigma and use of maternity services may be different for adolescent women. We tried to address these limitations by focusing our qualitative interviews on the needs and situation of women in these communities who do not use maternity services.

### Implications

These findings from the MAMAS Study indicate that health messaging for safe motherhood should stress that delivery in a health facility is recommended and important for all women, not just those with HIV or pregnancy complications. In addition, it is essential to ensure that services are welcoming and accessible for the poorest, least educated women, since they disproportionately deliver with unskilled TBAs [4]. Uptake of facility-based delivery is similar to other patterns of health care utilization globally, where the poorest, least educated, and socially marginalized feel mistrust of the health system [66].

By working to reduce HIV-related stigma in communities, families, couples, and health facilities, programs may increase pregnant women's health care utilization in high HIV prevalence settings. Our findings indicate that HIV-related stigma reduction interventions may increase not only utilization of PMTCT and HIV services, but also utilization of essential maternity services such as skilled attendance at delivery for all women regardless of their HIV serostatus. Combining known HIV stigma-reduction strategies [67]–[69] with maternal health and PMTCT interventions has the potential to reduce some of the most pressing health problems for women and children throughout sub-Saharan Africa.

## Abbreviations

ANC: antenatal clinic
MAMAS Study: Maternity in Migori and AIDS Stigma Study
PLWH: persons living with HIV
PMTCT: prevention of mother-to-child transmission
SD: standard deviation
TBA: traditional birth attendant
